# Troponin-I is present as an essential component of muscles in echinoderm larvae

**DOI:** 10.1038/srep43563

**Published:** 2017-03-08

**Authors:** Shunsuke Yaguchi, Junko Yaguchi, Hiroyuki Tanaka

**Affiliations:** 1 Shimoda Marine Research Center, University of Tsukuba, 5-10-1 Shimoda, Shizuoka 415-0025, Japan; 2 Faculty of Fisheries Sciences, Hokkaido University, 3-1-1 Minato-cho, Hakodate, Hokkaido 041-8611, Japan

## Abstract

The troponin complex, composed of Troponin-I, Troponin-T and Troponin-C, is an essential mediator of the contraction of striated muscle downstream of calcium signaling in almost all bilaterians. However, in echinoderms and hemichordates, collectively termed Ambulacraria, the components of the troponin complex have never been isolated, thus suggesting that these organisms lost the troponin system during evolution. Here, by analyzing genomic information from sea urchins, we identify the troponin-I gene and isolate its complete mRNA sequence. Using this information, we reveal that the larval muscles express this gene and its translated product and that the protein is definitely a functional molecule expressed in sea urchin larvae by showing that Troponin-I morphants are unable to swallow algae. We conclude that muscular contraction in all bilaterians universally depends on a regulatory system mediated by Troponin-I, which emerged in the common ancestor of bilaterians.

Muscle produces the main driving force necessary for animal behaviors such as walking, swimming, flying, and eating. The force in each contractile unit depends on the sliding actin-myosin assembly, which is highly conserved among eumetazoans^1^,^2^. Ca^2+^ signaling, troponin regulation and actomyosin contraction—the input, the mediator, and the output, respectively—are well conserved in almost all bilaterians^3^. However, it has been reported that echinoderms and hemichordates, collectively termed Ambulacraria, lack all components of the troponin complex, which is composed of Troponin-I (TnI), Troponin-T (TnT), and Troponin-C (TnC), thus raising the question of how the troponin systems emerged in the ancestors of bilaterians and how they evolved in the deuterostomes^4^,^5^,^6^. One possibility that has been suggested is that Ambulacraria has simply lost the troponin complex and uses another regulatory system, such as direct Ca^2+^ binding to myosin^6^,^7^. However, the genomic sequencing of *Strongylocentrotus purpuratus* has revealed incomplete troponin candidate genes^8^,^9^, thus leading us to reconsider whether echinoderms have the troponin complex.

## Results and Discussion

**Figure 1:**
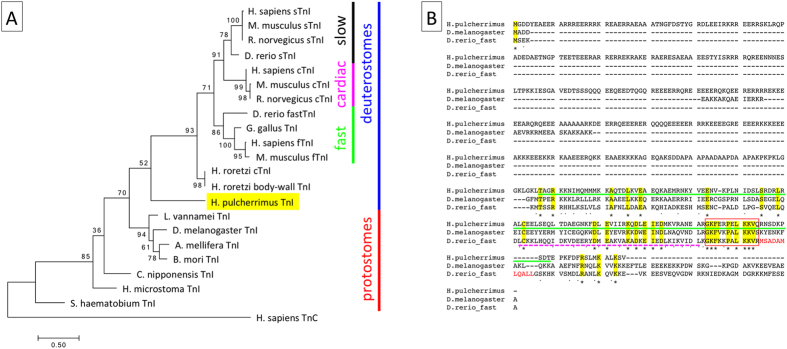
Sea urchin has a TnI gene. (**A**) The phylogenic tree of TnI based on the C-terminal troponin motif indicates that the sea urchin TnI forms a sister group with chordate TnI. The number at each branch point is the bootstrap value (n = 500). In vertebrates, TnI is categorized into three groups: slow, cardiac, and fast TnIs. *H. sapiens* troponin-C (TnC) was used as the outgroup. Accession numbers for each TnI are listed in the Table 1. The bar indicates evolutionary distance. (**B**) The alignment of TnI from sea urchin, fly and zebrafish. The red square indicates the inhibitory region. The magenta and green underlines indicate the positions of the TnT binding site and of the amino acid sequence equivalent to the position of the antigen of *Mesocentrotus nudus* TnI, respectively. The red characters in the zebrafish sequence indicate the switch region.

By analyzing the genomes and transcriptomes of *S. purpuratus* (http://www.echinobase.org)^9^,^10^ and *Hemicentrotus pulcherrimus* (Yaguchi S, *unpublished*), a candidate TnI gene was found in *H. pulcherrimus* (LC187281). The complete cDNA was isolated, and it encodes a clear troponin motif in the C-terminal region. These findings are supported by phylogenic analysis of the troponin motif (equivalent to amino acids 311–430 in *H. pulcherrimus* Troponin-I) using MUSCLE alignment^11^ and the maximum-likelihood method^12^ in MEGA software (Fig. 1A)^13^. Together, these data clearly indicated that echinoderms, at least the sea urchin group, contain TnI in their genomes and that sea urchin TnI (termed HpTnI hereafter) fills the evolutionary position between those of protostomes and chordates (Fig. 1A). This tree also implied that the common ancestor of bilaterians probably has protostome-type TnI and that chordates developed their own TnI after diversification from the common ancestor of chordates and echinoderms.

The primary structure of HpTnI and the alignment with fly and zebrafish TnI showed characteristics specific to HpTnI. One such characteristic is the long N-terminal extension, and another is the absence of a Troponin-C (TnC) binding-switch region, which is essential for initiating actin-myosin interaction in the presence of Ca^2+^ (red characters in Fig. 1B)^14^. Because the actin-binding domain, termed the inhibitory region (red square in Fig. 1B)^15^,^16^, and the Troponin-T binding site (magenta underline in Fig. 1B)^17^ are highly conserved among species including sea urchins (Fig. 1B), these two characteristics are distinctive features of HpTnI. The presence of an N-terminal extension is well conserved between protostome TnI and vertebrate cardiac TnI^18^, although the sequence and the length of the extension are somewhat variable. Among TnIs in chordates, only ascidian body-wall TnI and vertebrate skeletal TnI lack N-terminal extensions; therefore, this region is likely to be a remnant of the common evolutionary origin of this protein. The N-terminal extension of HpTnI is much longer than those of other TnIs (Fig. 1B). In invertebrates, the function of the N-terminal extension is infrequently studied, and it has been suggested that the N-terminal extension is not required for the basic function of TnI in Mollusca^19^. In *C. elegans*, the N-terminal extension of TnI is not important for muscle contraction but is important for worm locomotion, the detailed mechanisms of which remain unclear^18^. The function of the N-terminal extension of HpTnI has not yet been investigated, but it might be required for the conformation and/or function of the sea urchin-specific troponin complex because TnC is probably missing from sea urchin genomes^8^. TnC generally functions as a Ca^2+^ sensor in the troponin complex and plays an important role in triggering muscle contraction in most striated muscles^20^. Because the genome of *S. purpuratus* lacks a TnC gene, it is reasonable that HpTnI may lack a TnC-binding switch region in its polypeptide sequence. However, this deficiency makes it important to identify the mechanism for regulating muscle contraction via calcium signaling and TnI/TnT in sea urchins. Given the similarity of their amino acid sequences, Calmodulin (CaM) might be an alternative to TnC, because the Ca^2+^-binding EF-hands of TnC and CaM are well conserved^21^. Alternatively, the complex of TnI and TnT might function independently. In the genome of *S. purpuratus*, the TnT gene has been manually annotated (SPU_006532)^8^,^9^, and thus the gene structures suggest that sea urchin groups lack TnC only in the troponin complex. The isolation of the complete TnT from *H. pulcherrimus* is awaited, and biochemical analysis of HpTnI and HpTnT with and without CaM on muscular components would indicate how members of the sea urchin group use the troponin complex in regulating muscle contraction via Ca^2+^.

**Figure 2:**
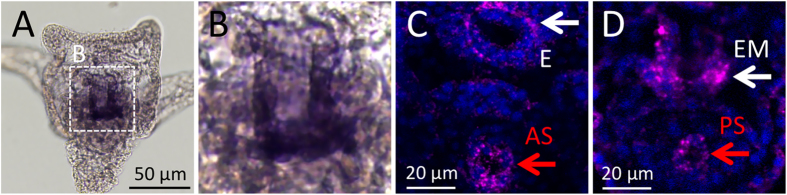
*In situ* hybridization of *HpTnI* in 3-day-old larvae. (**A**) *HpTnI* is likely expressed around the esophagus, but the signal is not clear from the chromogenic *in situ* hybridization. The region outlined by the dotted square is magnified in (**B**). (**C**,**D**) Fluorescence *in situ* hybridization revealed the detailed pattern of *HpTnI* expression in the esophagus (**E**), esophageal muscle (EM), anal sphincter (AS) and pyloric sphincter (PS).

**Figure 3:**
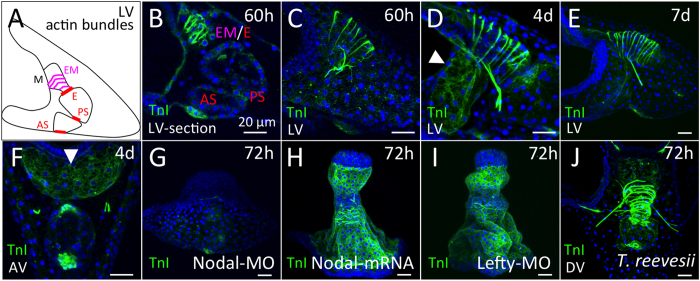
Expression of HpTnI protein in sea urchin larvae. (**A**) Schematic of muscular actin bundles in sea urchin larva. M, E, EM, PS, and AS indicate mouth, esophagus, esophageal muscles, pyloric sphincter and anal sphincter, respectively. (**B**) An optical section of a 60-h larva expressing HpTnI in the esophagus, esophageal region and the regions of the pyloric (PS) and anal (AS) sphincters. (**C**) Stacked image of a 60-h larva. HpTnI patterns are similar between 4 days (**D**) and 7 days (**E**). HpTnI expression in the ventral ectoderm is conspicuous by 4 days (**D**, arrowhead). (**F**) The anterior of the larva is at the top. HpTnI expression in the ventral ectoderm is clearly visible (arrowhead). (**G**) No ectodermal expression of HpTnI is visible in a Nodal morphant. (**H** and **I**) By contrast, with Nodal overexpression and in a Lefty morphant, the whole ectoderm except for the anterior and posterior ends expresses HpTnI. (**J**) A similar TnI pattern was observed in 72-h larvae of *Temnopleurus reevesii*. LV, lateral view; AV, anal view; DV, dorsal view. Bar = 20 *μ*m.

Next, we investigated whether the TnI gene is actually expressed in sea urchin larvae. Because the muscular components in larvae have been well reported^22^,^23^,^24^,^25^, TnI may be expressed in the same regions if the sea urchin TnI gene product is a truly functional element. In *S. purpuratus*, a temporal microarray analysis suggested that the incompletely annotated TnI gene (SPU_013183) is mainly expressed after gastrulation during embryogenesis^26^. *In situ* hybridization indicated that HpTnI-mRNA is expressed in the esophagus, the esophageal muscles and the pyloric and anal sphincters (Fig. 2A–D), corresponding to reports of the main muscles, as determined by the detection of actin bundles and myosin heavy chain (Fig. 3A)^24^. However, the 4-day larvae shrank so much during hybridization and the signal was so weak that mRNA in sphincters could be detected only by fluorescent *in situ* hybridization with confocal laser-scanning microscopy. Therefore, we developed an antibody against sea urchin TnI and used it to detect the endogenous protein in larvae. We tested the specificity of this antibody in a knockdown experiment described below. As with *HpTnI-*mRNA, HpTnI protein was present in the esophagus, the esophageal muscles and the pyloric and anal sphincters of 60-h larvae (Fig. 3B,C). These expression patterns in and around the digestive tract were unchanged for at least 1 week (Fig. 3D,E), thus suggesting that HpTnI functions in food consumption as one of the components of muscles. In addition to its expression in the above muscular regions, HpTnI was present in the ventral ectoderm (Fig. 3D,F, arrowheads), where we could not detect it by *in situ* hybridization. We attempted to confirm this expression by altering ectoderm regionalization. In Nodal morphants, in which the dorsal and ventral ectoderms are lost and most of the ectoderm is converted to a ciliary band, ectodermal HpTnI was absent (Fig. 3G). By contrast, in larvae overexpressing Nodal or in Lefty morphants, in which most of the ectoderm is converted to ventral ectoderm, HpTnI was expressed throughout the ectoderm, except in the animal plate and posterior ectoderm (Fig. 3H,I). These data clearly indicated that HpTnI is expressed in the ventral ectoderm. Because no muscular elements in the ventral ectoderm have been reported to date, the function of HpTnI in this region remains unclear. However, because the ectodermal region around the mouth shows dynamic movement during the swallowing of food, the epithelial tissue on the ventral side might contain muscular elements. Detailed observations with muscle-specific markers on this area in larval stages should reveal the full components of the ventral ectoderm.

To confirm whether the expression patterns of TnI are conserved among sea urchins, we investigated the protein expression patterns in another sea urchin, *Temnopleurus reevesii*^27^. As observed in *H. pulcherrimus*, TnI was expressed in the esophagus, the esophageal muscle and the pyloric and anal sphincters, as well as in the ventral ectoderm (Fig. 3J), thus indicating that TnI expression in muscles and ectodermal regions is evolutionarily conserved in the sea urchin group.

**Figure 4:**
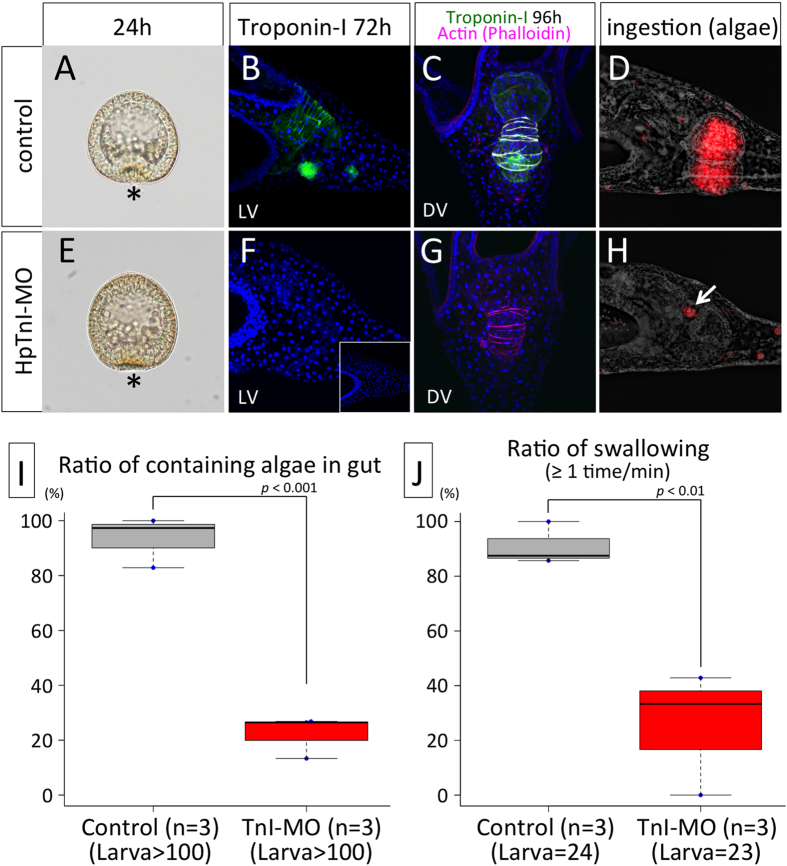
HpTnI is required for swallowing food. Gastrulation and actin bundling occur normally in the HpTnI morphant (**E**,**G**), as in the control (**A**,**C**). (**B**,**C**) HpTnI in a control larva. (**F**,**G**) The morphant lacks HpTnI protein. The fluorescence of algae is observed in the stomach and intestine in a control larva (**D**) but only in the throat in the morphant (**H**, arrow). (**I**) Ratios of algae ingestion in the control and HpTnI morphants. *p*: Student’s *t-test*. (**J**) Ratios of swallowing algae in the control and HpTnI morphants. *p*: Student’s *t-test*.

To investigate the function of HpTnI, we microinjected an anti-sense morpholino oligo against the HpTnI gene to block its translation and examined whether HpTnI morphants could swallow food. The timing of gastrulation was almost identical between control embryos and morphants (Fig. 4A,E), and morphants become pluteus larvae normally. At 72 h, the morphants lack most of the HpTnI protein (Fig. 4B,F). This was confirmed by using a second, nonoverlapping morpholino (Fig. 4F, inset). The absence of TnI protein signal in HpTnI morphants indicated the specificity of the antibody for HpTnI. By contrast, the actin fibers formed normally either with or without HpTnI (Fig. 4C,G), thus indicating that HpTnI is not necessary in the process of actin assembly. When we fed the alga *Chaetoceros calcitrans* to control and HpTnI-MO larvae, the morphants were unable to pass them into the stomach, whereas the control larvae were able to do so (Fig. 4D,H,I). To investigate whether the morphants lack the ability to swallow because of their TnI deficiency, we counted the number of swallowing behaviors under the microscope. The control larvae swallow at least once per minute (Fig. 4J, gray), whereas the swallowing rate of TnI morphants was significantly decreased (Fig. 4J, red). Given that the accumulation of algae in the throats of the morphants was observed (Fig. 4H, arrow), TnI is required for swallowing food in sea urchin larvae after the collection of food in the mouth. Although TnI generally functions as an inhibitor of muscle contraction, it remains unknown how the troponin complex and actomyosin bundles behave without TnI in sea urchin larva. Because cardiac TnI-knockout mice die within a month after birth because of impaired heart beating^28^, it is expected that in sea urchin larvae HpTnI-knockdown muscle cannot contract normally. Biochemical analysis of whole muscular components with or without HpTnI should reveal the detailed function of HpTnI in sea urchins.

For almost half a century, it has been reported that echinoderms have no troponin components, making it unclear how the regulation of muscle contraction was acquired during deuterostome evolution. The data shown here suggest that TnI was acquired in the bilaterian ancestors and has been retained through evolution until the present. We attribute the failure to detect TnI in echinoderms via biochemical methods, on which the previous papers depended, to the unexpected length of the N-terminal extension. The molecular weight of mammalian skeletal TnI is approximately 20 kDa, but that of HpTnI is approximately 52 kDa, which was unexpected until the complete mRNA was isolated in this study. Indeed, in Western blot using affinity-purified antibody, the molecular weight of HpTnI was over 52 kDa (Supplementary Figure S1). Three bands were detected in the blot, suggesting that the protein is modified via glycosylation and/or phosphorylation or that there are additional isoforms caused by the alternative splicing.

**Figure 5:**
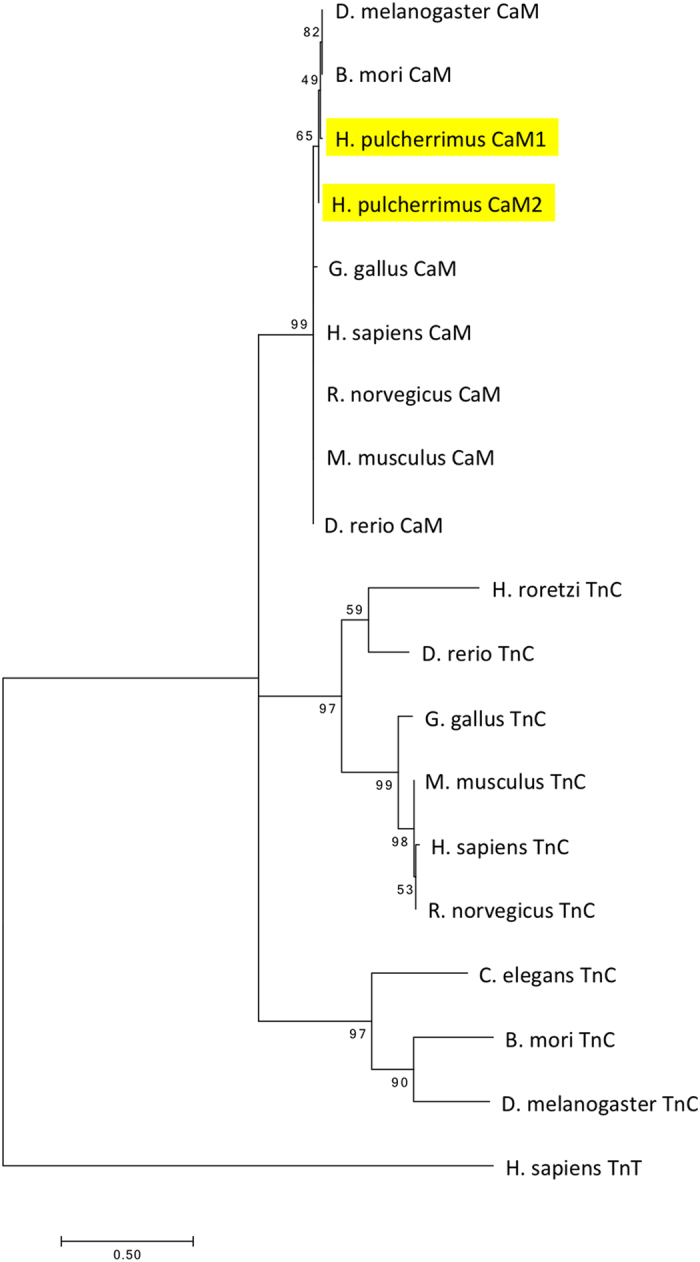
Phylogenic tree of Troponin-C (TnC) and Calmodulin (CaM). The number at each branch point is the bootstrap value (n = 500). *H. sapiens* Troponin-T was used as the outgroup. Accession numbers for each gene are listed in the Table 1. The bar indicates the evolutionary distance.

The absence of TnC in sea urchin genomes and of a TnC-binding switch region in HpTnI is of interest in considering the evolution of the troponin complex and the regulation of muscle contraction. Although the amino acid sequences of CaM and TnC are very similar, the phylogenic tree (Fig. 5) suggests that TnC was present in the ancestor of bilaterians and has been eliminated in Ambulacraria. The evolution of each component of the troponin complex should depend on its relationships with the other members of the complex. Thus, the absence of TnC and the lack of a TnC binding site in HpTnI should have coevolved. Because TnC is present in almost all reported bilaterians, the disappearance of TnC from Ambulacraria might be closely associated with the particularly long N-terminal extension of TnI in the sea urchin group.

The ultrastructural analysis demonstrated that the base of the esophagus is composed of striated muscle, but the esophageal muscle, the pyloric and anal sphincters are smooth muscles^22^. Because HpTnI is clearly present in all these muscles, it is suggested that the function of both striated and smooth muscles depends on Troponin-I-mediated regulation in the sea urchin larvae. Troponin systems are conventionally recognized as specific to striated muscle, but recent reports including our data suggest that they are present in all types of muscles of protostomes and vertebrates^29^,^30^. Thus, when troponin systems were acquired in the common ancestors of bilaterians, they were probably involved in the regulation of both striated and smooth muscles.

Like echinoderms, hemichordates have been reported to have no troponin complex^6^. However, TnT and TnC are computationally predicted in the genome of *Saccoglossus kowalevskii*. Although we did not include those data in the phylogenetic analysis in this paper because it remains unclear whether these genes are actually expressed and functional in hemichordates, the presence of these genes in genomes might support the idea that Ambulacraria have the troponin complex in muscles. Detailed biochemical analysis of Ambulacraria should provide insights into how the bilaterians acquired and evolved the troponin system for muscle contraction.

## Methods

### Animals and embryo culture

Adults of the sea urchins, *Hemicentrotus pulcherrimus* and *Temnopleurus reevesii,* were collected around Shimoda Marine Research Center, University of Tsukuba. The gametes were collected by the intrablastocoelic injection of 0.5 M KCl, and the *H. pulcherrimus* and *T. reevesii* embryos were cultured at 15 °C and 22 °C, respectively, in glass beakers or plastic dishes that contained filtered natural seawater (FSW) with 50 *μ*g/ml of kanamycin. To examine the food intake ability, we fed 5.0 *μ*l of Sunculture (algae, *Chaetoceros calcitrans* [Marinetech, Aichi, Japan]) to 20–100 individuals of 72-h larvae in 3.0 ml FSW for 30 min. The number of larvae whose stomach and intestine were filled with algae was counted, and the ratio was calculated from three independent batches (total number of larvae greater than 100). For each sample, the number of swallowing behaviors was counted under the microscope for one minute. Criteria for evaluating swallowing were based on the contraction of the esophagus.

### Antibody production

The *TnI* gene of *Mesocentrotus nudus* was partially cloned (LC187280), and the protein equivalent to amino acids 307–422 in *H. pulcherrimus* TnI (Fig. 1B, green underline) was produced in the vector pET-16b in *Escherichia coli* (strain BL21 Rosetta2 (DE3); Merck-Millipore, Darmstadt, Germany). The protein was purified with Toyopearl CM-650 M (Tosoh, Tokyo, Japan) and hydroxyapatite (Nacalai tesque, Kyoto, Japan) columns under conditions of 6 M urea, and it was then immunized to rabbits. The serum was collected and the specific antibody was purified with a TnI-conjugated Toyopearl AF-formyl-650 M (Tosoh) column. All experimental procedures using rabbits were performed according to the guidelines of and approved by the animal care committee of Hokkaido University (ID: 14–0073).

### Microinjection, whole-mount *in situ* hybridization and immunohistochemistry

These methods were described in detail previously^31^. The morpholino (Gene Tools, Philomath, OR, USA) sequences and the in-needle concentrations with 24% glycerol were as follows:

HpTnI-MO1 (0.5 mM): 5′-CTGCTTCATAGTCGTCACCCATAGT-3′,

HpTnI-MO2 (0.5 mM): 5′-TACCCTTCCAATTCAGGCCCTACAC-3′,

Nodal-MO (0.3 mM): 5′-AGATCCGATGAACGATGCATGGTTA-3′,

Lefty-MO (0.4 mM): 5′-AGCACCGAGTGATAATTCCATATTG-3′.

Nodal mRNA was synthesized from linearized plasmids using the mMessage mMachine kit (Thermo Fisher Scientific) and injected at 0.2 *μ*g/*μ*l in 24% glycerol in needles.

Dig-labeled RNA probe (0.4 ng/*μ*l final concentration) against the whole coding region of HpTnI was used for *in situ* hybridization. The sequence information of full-length HpTnI was provided by transcriptome analysis of *H. pulcherrimus*, which will be published elsewhere. The coding region was confirmed and isolated by the polymerase chain reaction using a cDNA pool of embryonic stages with KOD-FX (TOYOBO) and the following primers complementary to the 5′- and 3′-untranslated regions:

TnI-F1: 5′-GGAAGGGTAACTTCTCTGCTTAATTTTTAG

TnI-R1: 5′-GGAGACATTGGCACACGAATAAGGAGATGA.

In whole-mount immunohistochemistry, samples were blocked with 2.5% skim milk in PBS containing 0.1% Tween-20 for 1 h at RT and incubated overnight with TnI antibodies (1:100 dilution) at 4 °C. The samples were fixed with formaldehyde and washed with PBS (three 10-min washes) before treating rhodamine-phalloidine for 30 min to visualize the fibrous actin.

### Western blotting

Four-day larvae were stored at −80 °C until use. Each sample was dissolved in sodium dodecylsulfate (SDS)-sample buffer, approximately 10 larvae/*μ*l, separated by 10% SDS-polyacrylamide gel electrophoresis (SDS-PAGE) under reducing condition according to the methods of Porzio and Pearson^32^, and electrically transferred to nitrocellulose filters according to Towbin *et al*.^33^. The blot was blocked with 3.2% skim milk in TBS-T (20 mM Tris-HCl (pH 7.6), 0.15 M NaCl, 0.05% Tween-20) and incubated with anti-TnI antibody (1:2,500 dilution) overnight at room temperature. The primary antibody was detected with HRP-conjugated anti-rabbit antibody (1:5,000 dilution), and the immunoreaction was visualized using Trident femto-ECL (Gene Tex) and LAS-1000mini (Fujifilm).

## Additional Information

**How to cite this article:** Yaguchi, S. *et al*. Troponin-I is present as an essential component of muscles in echinoderm larvae. *Sci. Rep.* **7**, 43563; doi: 10.1038/srep43563 (2017).

**Publisher's note:** Springer Nature remains neutral with regard to jurisdictional claims in published maps and institutional affiliations.

## Supplementary Material

Supplementary Figure S1

## Figures and Tables

**Table 1 t1:** Gene ID list.

Name	Gene ID
Homo sapiens TnC	AAA91854
Apis mellifera TnI	NP_001035346
Chlamys nipponensis TnI	AE43658
Drosophila melanogaster TnI	CAA42020
Schistosoma haematobium TnI	XP_012800091
Hymenolepis microstoma TnI	CDS29158
Litopenaeus vannamei TnI	AFW99839
Bombyx mori TnI	NP_001037295
Rattus norvegicus cTnI	NP_058840
Homo sapiens cTnI	AAV38324
Danio rerio sTnI	NP_001008613
Danio rerio fTnI	NP_001009901
Homo sapiens fTnI	NP_001139301
Gallus gallus fTnI	NP_990748
Mus musculus fTnI	NP_033431
Homo sapiens sTnI	NP_003272
Rattus norvegicus sTnI	NP_001079781
Mus musculus sTnI	NP_001106173
Rattus norvegicus cTnI	NP_058840
Mus musculus cTnI	NP_033432
Halocynthia roretzi cTnI	BAB83811
Halocynthia roretzi body-wallTnI	BAB83810
Bombyx mori TnC	NP_001037594
Drosophila melanogaster TnC	CAA53628
Halocynthia roretzi TnC	BAA13631
Homo sapiens TnT	AAB30272
Gallus gallus TnC	AAA49097
Caenorhabditis elegans TnC	BAB84566
Rattus norvegicus TnC	NP_001032428
Mus musculus TnC	NP_033420
Danio rerio TnC	AAO50211
Homo sapiens CaM	AAD45181
Rattus norvegicus CaM	NP_114175
Mus musculus CaM	NP_031615
Gallus gallus CaM	NP_990336
Danio rerio CaM	NP_999901
Bombyx mori CaM	NP_001040234
Drosophila melanogaster CaM	NP_523710
Hemicentrotus pulcherrimus CaM1	LC187282
Hemicentrotus pulcherrimus CaM2	LC187283
