# Supplementary material for: Troponin-I is present as an essential component of muscles in echinoderm larvae
Source: Sci Rep. 2017 Mar 8;7:43563. doi: 10.1038/srep43563 (PMC5341096; doi:10.1038/srep43563)
Supplement: Supplementary Figure S1 [file srep43563-s1.pdf]

Troponin-I is present as an essential component of muscles in echinoderm larvae

Shunsuke Yaguchi[1,†,*], Junko Yaguchi[1,†], Hiroyuki Tanaka[2]

[1]Shimoda Marine Research Center, University of Tsukuba, 5-10-1 Shimoda, Shizuoka 415-0025, Japan

[2]Faculty of Fisheries Sciences, Hokkaido University, 3-1-1 Minato-cho, Hakodate, Hokkaido 041-8611, Japan

[†]The first two authors contributed equally.

Short title: Troponin-I in echinoderm.

*Corresponding Author: Shunsuke Yaguchi, Shimoda Marine Research Center, University of Tsukuba, 5-10-1, Shimoda, Shizuoka, Japan 415-0025

Phone: +81-558-22-6716

Fax: +81-558-22-0346

E-mail: yag@shimoda.tsukuba.ac.jp

Supplementary Figure S1.

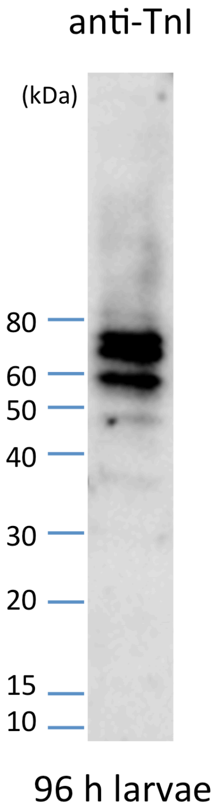

Western blot showing the molecular mass of HpTroponin-I. Three strong bands were detected between 55-75kDa.
